# Biosurfactant and biopolymer producing microorganisms from West Kazakhstan oilfield

**DOI:** 10.1038/s41598-024-52906-7

**Published:** 2024-01-27

**Authors:** Ulzhan Shaimerdenova, Gulzhan Kaiyrmanova, Wioleta Lewandowska, Marek Bartoszewicz, Izabela Swiecicka, Aliya Yernazarova

**Affiliations:** 1 Faculty of Biology and Biotechnology, Al-Farabi Kazakh National University, 71 Al-Farabi Ave, 050038 Almaty, Kazakhstan; 2 Doctoral School of Exact and Natural Sciences, University of Białystok, 1K Konstanty Ciołkowski Str, 15-245 Białystok, Poland; 3 Faculty of Biology, University of Bialystok, 1J Konstanty Ciołkowski Str, 15-245 Bialystok, Poland; 4 Laboratory of Applied Microbiology, Faculty of Biology, University of Bialystok, 1J Konstanty Ciołkowski Str, 15-245 Bialystok, Poland

**Keywords:** Biotechnology, Microbiology

## Abstract

Microbiological enhanced oil recovery (MEOR) uses indigenous or exogenous microorganisms and nutrients to enhance oil production through synthesis of metabolites reducing oil viscosity and surface tension. In order to find bacteria suitable for MEOR, we studied 26 isolates from wells in the Akingen oilfield in West Kazakhstan. Six of them were selected for further analysis based on their ability to reduce surface tension to less than 40 mN/m, with the A9 isolate exhibiting tension reduction values of 32.76 ± 0.3 mN/m. Based on the morphological features, biochemical activities, and the 16S rRNA gene, the isolates were classified to the *Bacillus subtilis* group. In the phylogenetic analysis the isolates grouped into two main clusters. Genes encoding the surfactin synthetase subunits were found in A2, A8, A9, A12*,* PW2, only the PW2 strain had *lchAA* encoding lichenysin, while *sacB* encoding levan was noted in A2, A8, A9, and A12. The expression of *srfAB*, *srfAC*, and *sacB* tested with qPCR varied among strains. Nevertheless, whereas temperature moderately affects the expression level, with the highest level recorded at 40 °C, salinity significantly impacts the expression of the genes encoding biosurfactants*. B. subtilis* strains isolated in the study, especially A9, are promising for microbial-enhanced oil recovery.

## Introduction

The high worldwide demand for crude oil led to enhanced oil recovery and more effective oil extraction from reservoirs. However, among the tertiary methods of oil recovery enhancement, implementing microorganisms is of great interest. This approach, called microbiological enhanced oil recovery (MEOR), has several advantages, such as environmental safety and an economically low cost^1,2^. MEOR technologies use both, microorganisms and their metabolites, including biosurfactants, biopolymers, bioacids, solvents, biogas, and enzymes, to produce residual oil^3,4^. Nevertheless, biosurfactants stand out promisingly among metabolites with the highest prospects for use in MEOR^5^.

Microorganisms can produce various biosurfactants that can replace chemical analog compounds. Biosurfactants are amphiphilic compounds with a hydrophilic end and a hydrophobic end, separated at the interface between different phases^6,7^. In addition, from the perspective of MEOR applications, biosurfactants have many advantages, such as biodegradability, low toxicity, low critical micelle concentration (CMC), and the ability to be obtained from renewable and cheap substrates^8,9^. Biosurfactants are known for their excellent surface activity, which also includes reducing the surface and interfacial tension between different phases (liquid–air, liquid–liquid, and liquid–solid). The ability to reduce surface and interfacial tension is achieved due to the adsorption of biosurfactants at different phases, which leads to more significant interaction and mixing of dissimilar phases. Consequently, they have an emulsifying activity that can effectively emulsify two immiscible liquids, such as hydrocarbons or other hydrophobic substrates^10^. Biosurfactants have a wide range of biotechnological applications in the oil industry. They work better than chemical analogs due to their higher compatibility with the environment, reduced toxicity, and the ability to be produced from renewable sources. Biosurfactants mainly contribute to reducing the interfacial tension between oil-bearing rocks and oil solution, changing the wettability of porous media, emulsifying crude oil, and affecting the oil, making it even more fluid-like^11,12^.

Microorganisms synthesize various types of biosurfactants, which are classified based on their chemical composition and microbial origin, that includes glycolipids and lipopeptides, lipoproteins, phospholipids and fatty acids, polymeric surfactants, and particulate surfactants^13^. Glycolipids and lipopeptides are low-molecular biosurfactants that reduce surface and interfacial tension, while other high-molecular biosurfactants (e.g., lipoproteins, lipopolysaccharides, and amphipathic polysaccharides) are effective in stabilizing emulsions^14,15^. *Pseudomonas* spp., *Bacillus* spp., *Rhodococcus* spp., *Candida* spp., and *Acinetobacter* spp. are among the well-known bacterial manufacturers of biosurfactants^16–20^. When applying MEOR associated with the production of metabolic products, it is necessary to consider some factors that significantly affect the results of the process (geological and physical properties of the reservoir, including temperature, pressure, salinity, pH, and composition of reservoir water)^2,21^.

**Figure 1:**
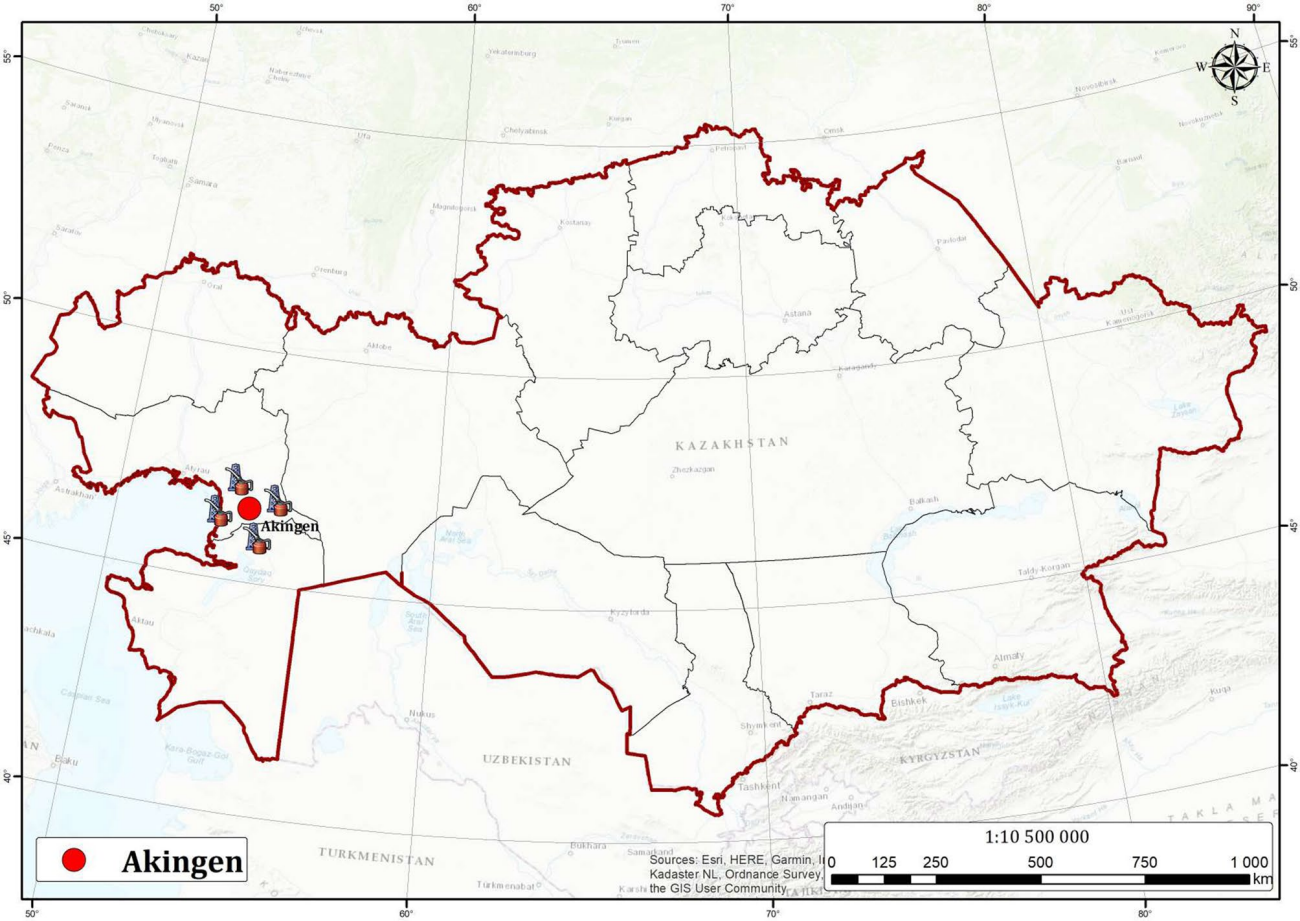
The location of the Akingen oilfield in Kazakhstan where the samples (No.302, No.329 oil wells and block cluster pumping station) were collected. The map was created using Arcgis 10.4.

In the context of MEOR, this study focused on screening and studying bacteria producing biosurfactants from the formation water of Akingen oil reservoirs in Western Kazakhstan (Fig. 1). The selected bacteria, aligned with MEOR criteria, were tested for biosurfactant and biopolymer production, and their phylogenetic relationship. Additionally, the study evaluated the presence of biosurfactant and biopolymer genes and their expression under different conditions. The relevance of this research is underscored by challenges posed in oil fields, including high water cut (80–90%), significant untapped oil reserves (up to 60–70%) in deep reservoirs, high oil viscosity, low reservoir permeability, and complex geological structures in the oil fields in Kazakhstan. Moreover, in Kazakhstan, most of the hydrocarbon deposits have already been discovered and commercially produced. In this regard, the residual oil left behind in these mature hydrocarbon deposits after primary and secondary oil recovery offers an opportunity to implement EOR processes, including the application of microbial-enhanced oil recovery (MEOR) technology.

## Results

### Oil wells screening for possible MEOR process and biosurfactant and biopolymer-producing bacteria

We started searching for bacteria with potential usage in MEOR with a chemical analysis of the formation waters of two oil wells (No. 302, No. 329) and the block cluster pumping station (BCPS) from West Kazakhstan (Fig. 1). Overall, we tested 18 chemical parameters, which results are given in Table 1. For instance, the salinity of the formation water ranged from 111.8 to 148 g/l, its temperature varied from 35 to 41 °C, while pH ranged from 6.39 to 6.65. Therefore, the oil wells chosen for the study are applicable for MEOR, since they showed optimal parameters for bacterial growth.

**Table 1:** Chemical parameters of formation water collected in the Akingen oilfield in Western Kazakhstan.

Parameter	BCPS output ^a, b^	Oil well No. 302	Oil well No. 329
HCO_3_^−^ (mg/dm^3^)	161.65	158.60	137.25
Cl^−^ (mg/dm^3^)	90 849.49	80 161.31	68 582.46
Ca^2+^ (mg/dm^3^)	3 406.80	3 106.20	3 006.00
Mg^2+^ (mg/dm^3^)	1 672.00	1 641.60	1 094.40
Na^+^ + К^+^ (mg/dm^3^)	51 931.70	45 398.55	39 028.24
Fe^3+^ (mg/dm^3^)	23.80	4.48	7.56
Fe^2+^ (mg/dm^3^)	4.34	12.60	N.D
CO_3_^−^ (mg/dm^3^)	N.D	N.D	N.D
SO_4_^2−^ (mg/dm^3^)	N.D	N.D	N.D
J^−^ (mg/dm^3^)	2.94	1.68	2.10
Br^−^ (mg/dm^3^)	128.18	112.58	103.22
NO_3_—(mg/dm^3^)	0.438	0.540	0.865
Dissolved O_2_ (mg/dm^3^)	4.88	2.28	4.92
Salinity (g/l)	148	130.5	111.8
Density at 20 °C (g/cm^3^)	1.1002	1.0891	1.0759
pH	6.40	6.39	6.65
Kinematic viscosity (mm^2^/s)	1.27	1.21	1.195
Temperature (°C)	35–40	37–41	–^c^

^a^BCPS: block cluster pumping station.

^b^N.D.: not detected.

^c^Ambient temperature depending on time of sample collection.

At the initial step, we isolated 26 bacteria from different samples. The bacteria were investigated in order to identify potential biosurfactants producers with the use of (i) the oil spreading test, (ii) the emulsifying activity, and (iii) stalagmometric methods. The results of these tests are listed in Table 2. All bacteria showed a positive result in the oil spreading test with a clear zone that varied from 0.7 ± 0.1 to 2.9 ± 0,1 cm. The emulsification index fluctuated from 26.2 ± 0.3 to 66.5 ± 0.4%, with the highest emulsification index observed for the A12 isolate. The control emulsification index was 74.2%. Surface tension tested by using Traube’s stalagmometer varied from 51.2 ± 0.3 to 68.7 ± 0.2 mN/m. The most active isolates, A2, A4, A8, A9, A11, A12, R1, R4, PW2, demonstrated (i) a clear zone area above 2.6 cm in the oil spreading, (ii) an emulsification index above 60%, and (iii) surface tension below 66 mN/m. These isolates were selected for further study (for details see Table 2).

**Table 2:** Screening results of biosurfactant-producing bacteria from water samples from the Akingen oilfield located in Western Kazakhstan with potential use in MEOR.

Isolate^a^	Oil spreading test (cm)	Emulsifying index (%)	Surface tension (mN/m)
A1	1.1 ± 0.1	42.6 ± 0.5	68.6 ± 0.5
A2*	2.7 ± 0.1	62.6 ± 0.5	64.2 ± 0.4
A3	0.7 ± 0.1	33.1 ± 0.2	68.6 ± 0.5
A4*	2.6 ± 0.1	65.3 ± 0.5	60.3 ± 0.5
A5	2.0 ± 0.2	58.2 ± 0.2	68.0 ± 0.1
A6	1.3 ± 0.3	41.2 ± 0.4	67.1 ± 0.2
A7	1.1 ± 0.2	51.1 ± 0.2	66.5 ± 0.4
A8*	2.6 ± 0.1	61.2 ± 0.1	66.0 ± 0.1
A9*	2.8 ± 0.1	60.1 ± 0.1	51.2 ± 0.3
A10	1.1 ± 0.1	34.1 ± 0.1	68.6 ± 0.5
A11*	2.6 ± 0.1	65.5 ± 0.4	65.8 ± 0.3
A12*	2.9 ± 0.1	66.5 ± 0.4	65.7 ± 0.4
A13	1.8 ± 0.3	44.3 ± 0.2	67.3 ± 0.2
A14	0.9 ± 0.1	41.2 ± 0.4	66.5 ± 0.4
R1*	2.7 ± 0.1	60.2 ± 0.4	66.0 ± 0.2
R2	2.2 ± 0.2	55.4 ± 0.2	68.3 ± 0.5
R3	2.2 ± 0.1	45.2 ± 0.4	68.6 ± 0.5
R4*	2.8 ± 0.1	60.3 ± 0.3	64.2 ± 0.4
R5	2.0 ± 0.1	41.6 ± 0.1	68.6 ± 0.5
R6	1.6 ± 0.2	33.1 ± 0.2	68.7 ± 0.2
R7	1.6 ± 0.1	36.0 ± 0.1	68.0 ± 0.1
R8	2.3 ± 0.1	42.1 ± 0.1	68.1 ± 0.2
R9	0.8 ± 0.3	26.2 ± 0.3	67.3 ± 0.2
R10	1.7 ± 0.6	58.1 ± 0.2	68.1 ± 0.1
PW1	1.6 ± 0.1	44.0 ± 0.1	67.1 ± 0.2
PW2*	2.9 ± 0.2	61.2 ± 0.3	64.0 ± 0.1
1% Tween-20	4.8 ± 0.3	74.2 ± 0.3	52.7 ± 0.6

^a^The most active isolates are indicated with a star.

**Figure 2:**
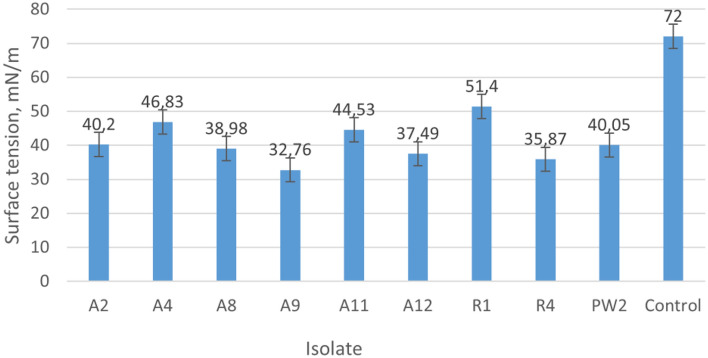
Surface tension measurement of biosurfactant-producing bacteria from water samples from the Akingen oilfield located in Western Kazakhstan.

The selected bacteria were also tested for the surface tension in the ST1 power tensiometer. As shown in Fig. 2, the surface tension of our isolates was in the range of 32.76 ± 0.3–51.40 ± 0.4 mN/m, while the surface tension of distilled water used as a control was 72 ± 0.1 mN/m. The most promising surface tension (32.76 ± 0.3 mN/m) was obtained with the A9 isolate. From the nine bacteria chosen for this analysis based on the screening biosurfactant-bacteria (Table 2), six isolates, e.g. A2, A8, A9, A12, R4, and PW2, which showed the best results for the surface tension (< 40 mN/m), were selected into the identification study.

### Identification of biosurfactant-producing bacteria and phylogenetic analysis

**Figure 3:**
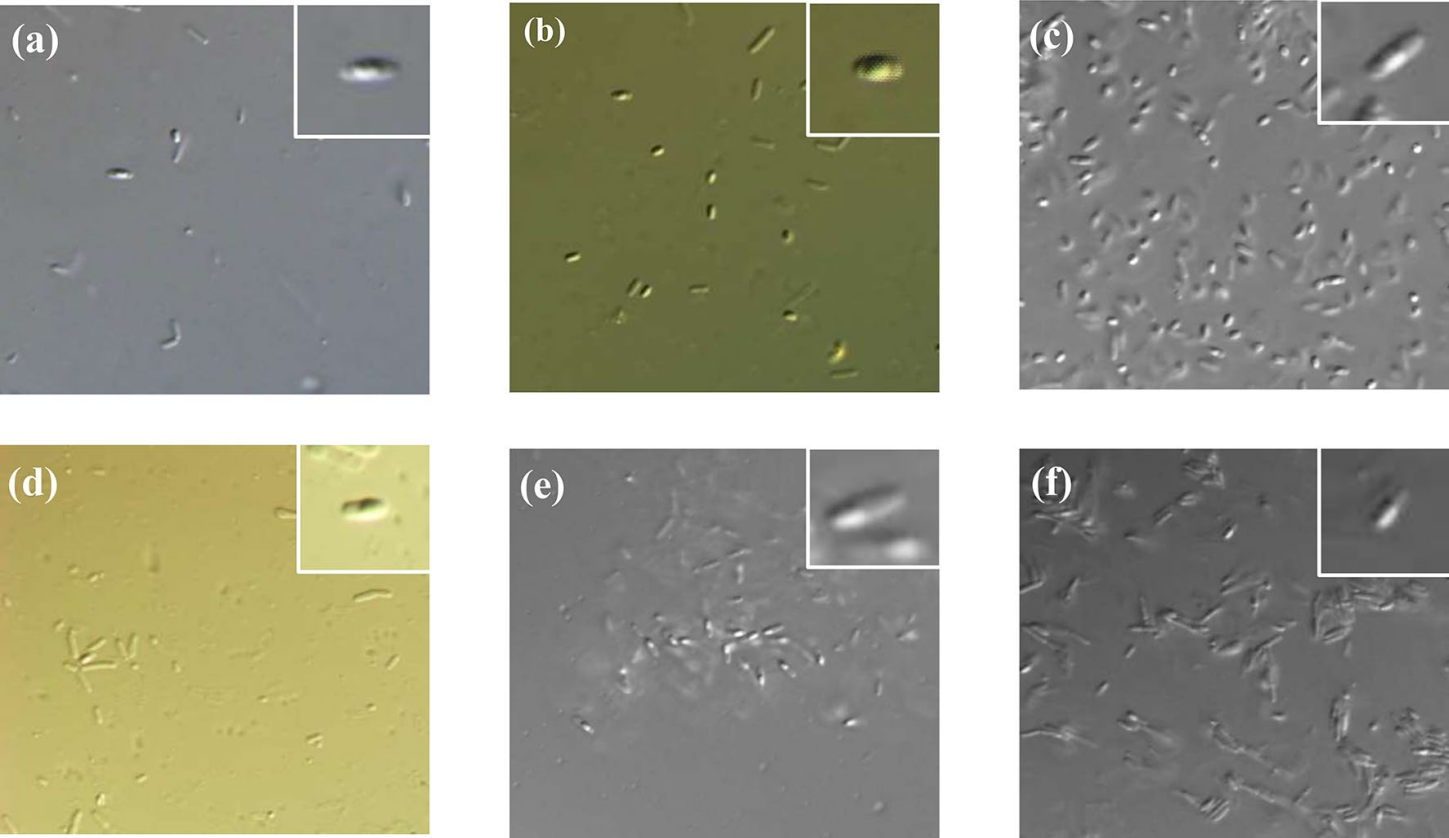
Photomicrographs of isolates from formation water samples (the Akingen oilfield located in Western Kazakhstan) viewed by phase-contrast microscope. (**a**) A2 with ellipsoidal, centrally located spores; (**b**) A8 with ellipsoidal, central spores; (**c**) A9 with ellipsoidal, subterminal spores; (**d**) A12 with ellipsoidal, subterminal spores; (**e**) R4 with ellipsoidal, subterminal spores; (**f**) PW2 with ellipsoidal, subterminal spores. All strains were cultivated in nutrient agar for 48 h at 40 °C. The photos were taken with the Olympus BX61 at the magnification of 1000x.

All isolates selected for potential usage in MEOR grew well in the presence of oxygen, were Gram-positive, and formed ellipsoidal endospores. These signatures allowed to classify the isolates into the genus *Bacillus* (Fig. 3).

The biochemical profiles of the oil-associated isolates determined with API tests strongly indicated the belonging of the oil-associated isolates to the *B. subtilis* group. For instance, all isolates decomposed 13 compounds (L-arabinose, D-ribose, D-glucose, D-fructose, D-mannose, D-mannitol, methyl-α-D-glucopyranoside, arbutin, esculin, salicin, D-cellobiose, D-saccharose, D-trehalose), whereas 21 carbohydrates (erythritol, D-arabinose, L-xylose, D-adonitol, methyl-β-D-xylopyranoside, L-sorbose, dulcitol, D-sorbitol, methyl-α-D-mannopyranoside, N-acetylglicosamine, D-melezitose, xylitol, gentiobiose, D-lyxose, D-fucose, L-fucose, D-arabitol, L-arabitol, potassium gluconate, potassium 2-ketogluconate, potassium 5-ketogluconate) were not broken down by these bacteria. Moreover, ten compounds (glycerol, D-xylose, inositol, amygladin, D-maltose, D-melibiose, D-raffinose, starch, glycogen, D-tagatose) were fermented by five strains but not by the A2 isolate. In turn, only A12 degraded D-lactose. Some isolates (A8, A9, and R4) decomposed inulin and D-turanose, while R4 and PW2 fermented D-galactose. No biochemical differences between the isolates were detected in 11 trials of API 20E used in the study. All isolates hydrolyzed β-galactosidase (ONPG test) and liquefied gelatin, but neither decarboxylated L-arginine, L-lysine nor L-ornithine. Moreover, all isolates were negative in citrate utilization, H_2_S production, urease activity, tryptophan deaminasation, and indole production. Furthermore, only three isolates (A12, R4, and PW2) produced acetoin in the Voges Proskauer test. Detailed results on the biochemical activity in API tests are given in Supplementary material Table S2.

**Figure 4:**
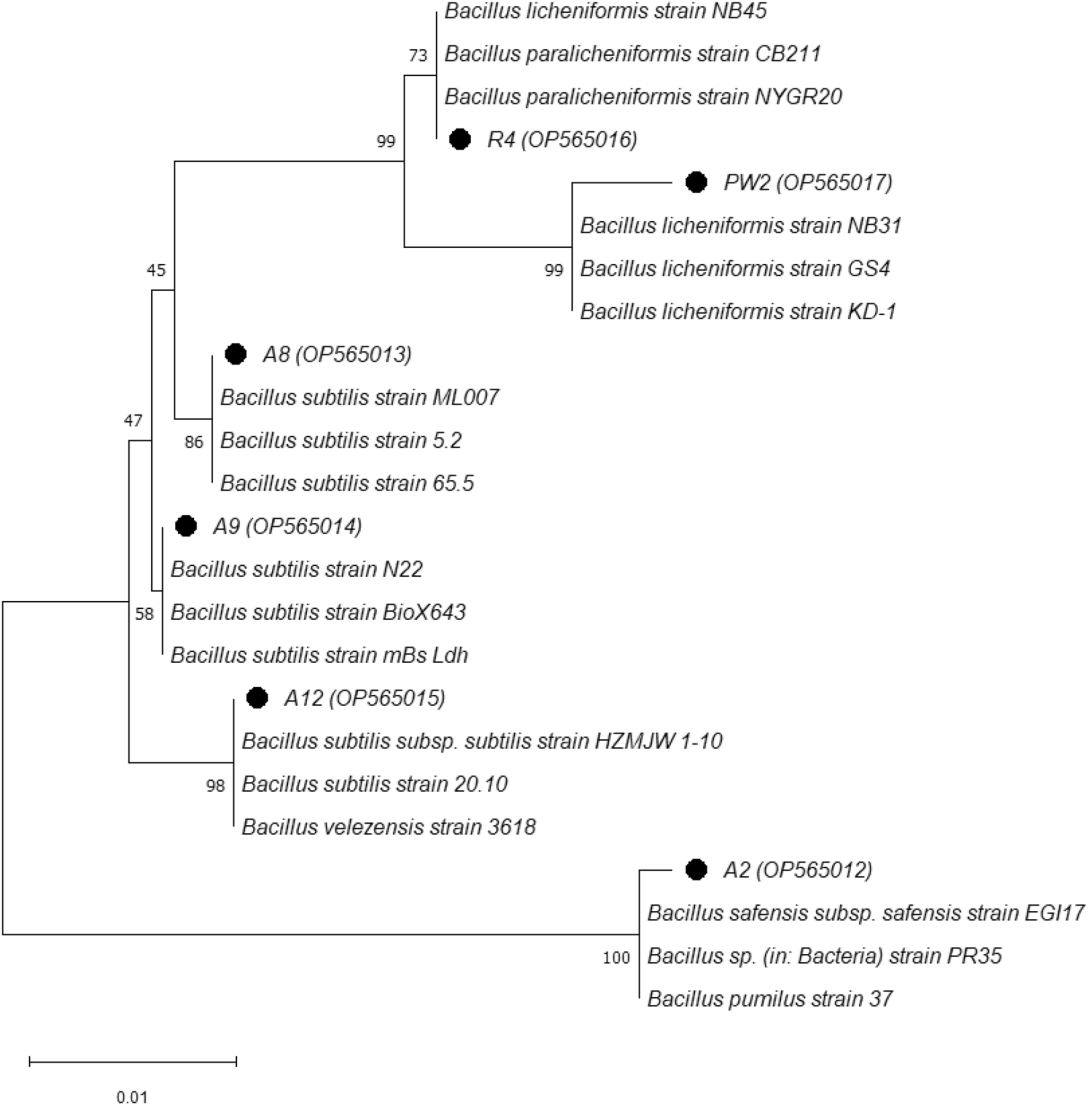
Phylogenetic tree of isolated microorganisms and some representatives of *Bacillus* spp. The percentage of replicate trees in which the associated taxa clustered together in the bootstrap test (1000 replicates) are shown next to the branches^76^. The sequence numbers in GenBank are shown in parentheses. The tree is drawn to scale, with branch lengths in the same units as those of the evolutionary distances used to infer the phylogenetic tree. The evolutionary distances were computed using the p-distance method and are in the units of the number of base differences per site. All positions containing gaps and missing data were eliminated (complete deletion option). There was a total of 619 positions in the final dataset.

In order to determine the species classification of the isolates with the potential usage in MEOR (for details see the first paragraph of Results), which were preliminary classified into the *B. subtilis* group, we performed genotyping at the conservative 16S rRNA locus. Based on the analysis of the nucleotide sequence of the 16S rDNA gene obtained, the isolates were classified as follows: *Bacillus safensis* (one strain), *Bacillus paralicheniformis* (one strain), *Bacillus licheniformis* (one strain), and *Bacillus subtilis* (three strains). The nucleotide sequences of 16S rRNA gene fragments were deposited in GenBank (http://www.ncbi.nlm.nih.gov). Detailed information on species identification is presented in Table 3. Additionally we constructed the phylogenetic trees using the nucleotide sequences of the 16S rDNA gene of the isolated bacteria (n = 6) and 18 nucleotide sequences of the representative strains of the genus *Bacillus* available in GenBank (Fig. 4). The phylogenetic tree is clearly divided into two clusters: (i) cluster 1 contains the A2 strain, which is more closely related to *Bacillus safensis* subp. *safensis*, and (ii) cluster 2 divided into subclusters, which include the remaining five strains. All *Bacillus* spp. strains from these two clusters showed a more than 99% similarity in the 16S rDNA gene sequence, indicating a closer relationship with the references.

**Table 3:** The species classification of biosurfactants-producing bacteria from water samples from the Akingen oilfield located in Western Kazakhstan.

Isolate	Accession no. of the closest sequence in GenBank	)	Classification ^a^
A2	MN704393.1	99.85	*Bacillus safensis* subsp*. safensis*
A8	MT636460.1	99.85	*Bacillus subtilis*
A9	ON795918.1	100	*Bacillus subtilis*
A12	MT605412.1	100	*Bacillus subtilis* subsp*. subtilis*
R4	MN922812.1	99.58	*Bacillus paralicheniformis*
PW2	MT214222.1	99.15	*Bacillus licheniformis*

^a^GenBank accession No. of the strains are given in the paragraph entitled Repositories.

### The presence and expression of the biosurfactant and biopolymer genes in the Bacillus subtilis group isolates

*Bacillus* spp. isolated from the Akingen oilfield were tested for the presence of the surfactin operon (*srfAA*, *srfAB*, *srfAC*, *srfAD*), that encodes the subunits of the surfactin synthetase. All four genes were found in two *B. subtilis* strains (A8 and A9), while in the A12 isolate the *srfAA*, *srfAC*, and *srfAD* genes were noted (Table 4). In turn, the *lchAA* gene encoding lichenysin was detected in *B. licheniformis* PW2 only (Table 4). Meanwhile the *sacB* gene encodes levan in four out of six strains, e.g. *B. safensis subsp. safensis* A2*, B. subtilis* A8, *B. subtilis* A9, and *B. subtilis* subsp. *subtilis* A12. Any of the tested genes was noted in *B. paralicheniformis* R4, while all six genes were identified in *B. subtilis* A9. The results of PCR screening are presented in Table 4.

**Table 4:** Presence of genes encoding biosurfactants (*srfAA, srfAB, srfAC, srfAD, lchAA*) and biopolymer (*sacB*) in the *B. subtilis* group isolates from the Akingen oilfield located in Western Kazakhstan.

Isolate	Gene^*a*^					
	*srfAA*	*srfAB*	*srfAC*	*srfAD*	*lchAA*	*sacB*
A2	−	−	−	−	−	+
A8	+	+	+	+	−	+
A9	+	+	+	+	−	+
A12	+	−	+	+	−	+
R4	−	−	−	−	−	−
PW2	+	−	−	−	+	−

^*a*^+, detected; −, not detected.

**Figure 5:**
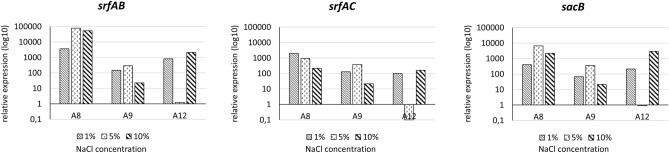
The changes in relative expression of the *srfAB, srfAC* and *sacB* genes in media supplemented with 1%, 5% and 10% NaCl in comparison to basic brain–heart infusion medium among *B. subtilis* A8, A9 and A12 isolates.

The relative expression of the biosurfactants genes among the *Bacillus subtilis* group isolates in the present study were assessed in three approaches: (i) comparing the *srfAB*, *srfAC*, and *sacB* relative expression in *B. subtilis* A9, and A12 in relation to *B. subtilis* A8, (ii) comparing changes in the relative genes expression in the isolates growing under different concentration of NaCl, and (iii) assessing changes in the relative expression of the genes in the isolates growing at different temperatures. First, it was found that the expression of the *srfAB*, *srfAC*, and *sacB* genes differs between tested isolates. The lowest relative expression levels of the *sfrAB* and *srfAC* genes were observed for *B. subtilis* A8, and the *sacB* gene for *B. subtilis* A12. In all cases, the highest relative expression was noted for *B. subtilis* A9 (Table 5). Secondly, comparative analysis of the *srf* operon and the *sacB* gene expression at different temperatures indicated moderate differences. The highest level of the *sacB* expression was noted at a temperature of 40 °C, and decreased at a temperature of 20 °C, resulting in a fivefold decrease in expression (in strain A12). The optimal temperature for the expression of the *srfAB* gene for all strains is 20 °C, and for the *srfAC* gene, mostly 40 °C. Interestingly, the A9 strain showed 1.8-fold higher expression of *srfAB* at 50 °C and 8.9-fold higher at 30 °C when compared to expression at 40 °C. For the *srfAC* the relative expression noted for this isolate at 20 °C was even 5.6-fold higher when compare to the expression at 40 °C. Thirdly, it was found that increasing the salinity to 1% results in up to 3,604-fold increase in the expression of *srfAB*, while 5% salinity results in even 78,218-fold increase in the expression of this gene. Higher salinity, however, does not result in a further increase in the *srfAB* expression. Only the A12 isolate displayed reduced expression of *srfAC* in a medium supplemented with 5% NaCl. Similarly, only this isolate responded with a decrease in expression to 5% salt concentration for *sacB*, while the other strains showed increases in the range of 357.6–6,636.0-fold (Fig. 5).

**Table 5:** Changes in relative gene expression among tested strains given as multiplicity of the expression of particular genes calculated for *B. subtilis* A8 isolate.

Isolate	Gene		
	*srfAB*	*srfAC*	*sacB*
A9	57,606.7	191.1	3.50
A12	37.63	86.21	0.03

### Crude biosurfactant production and Fourier transformed-infrared (FT-IR) spectrum

**Figure 6:**
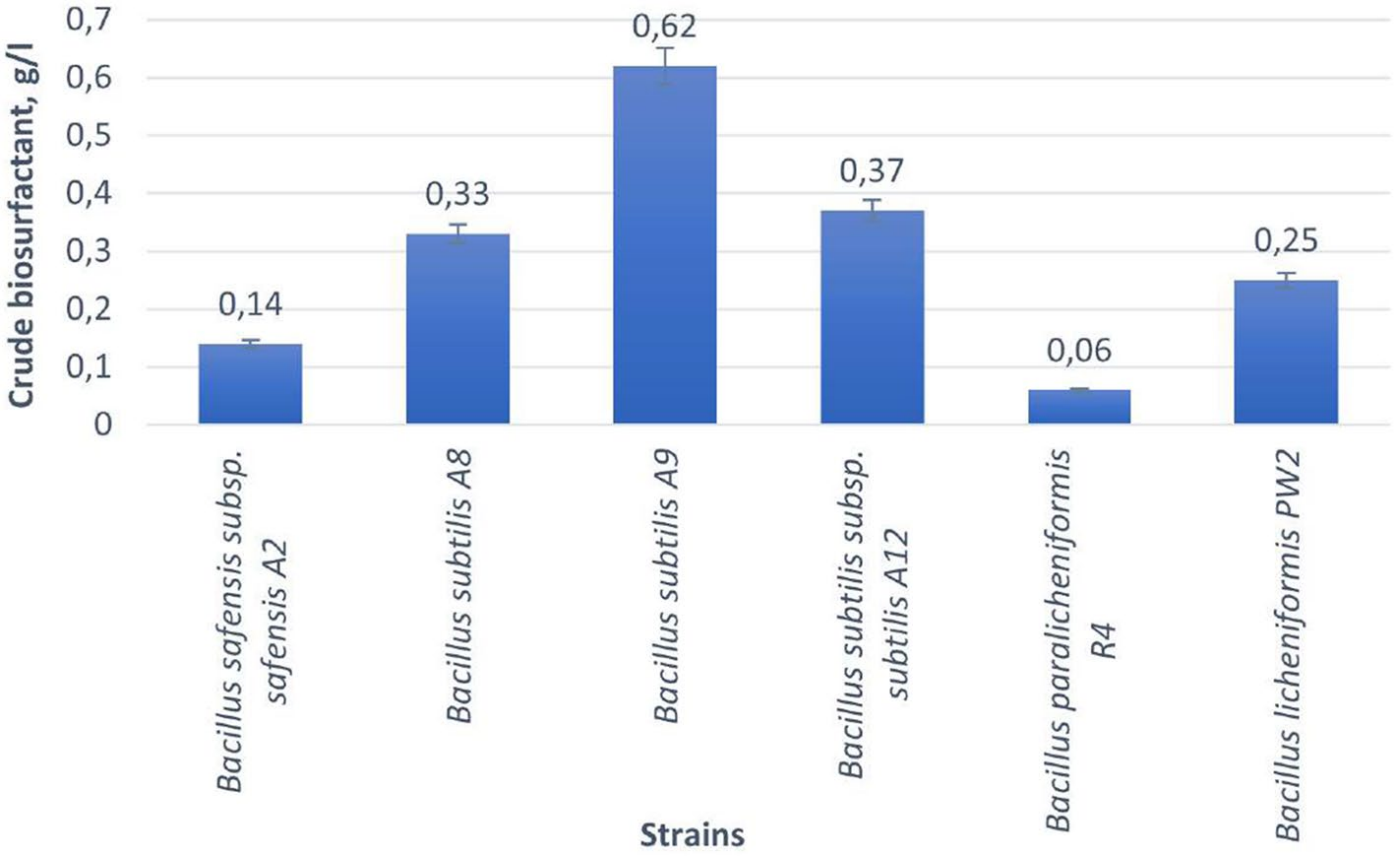
Yield of crude biosurfactants from *Bacillus* spp. strains from the Akingen field in West Kazakhstan.

This study presents data on the use of crude oil as a carbon source for biosurfactant production employing different strains of *Bacillus* spp. bacteria (Fig. 6). As illustrated in Fig. 6, all *Bacillus* spp. strains exhibited productivity in crude biosurfactant production, with *Bacillus subtilis* A9 demonstrating exceptionally high performance at 0.62 g/L. The lowest biosurfactant yield was observed in the strain *Bacillus paralicheniformis* R4, with a production level of 0.06 g/L.

**Figure 7:**
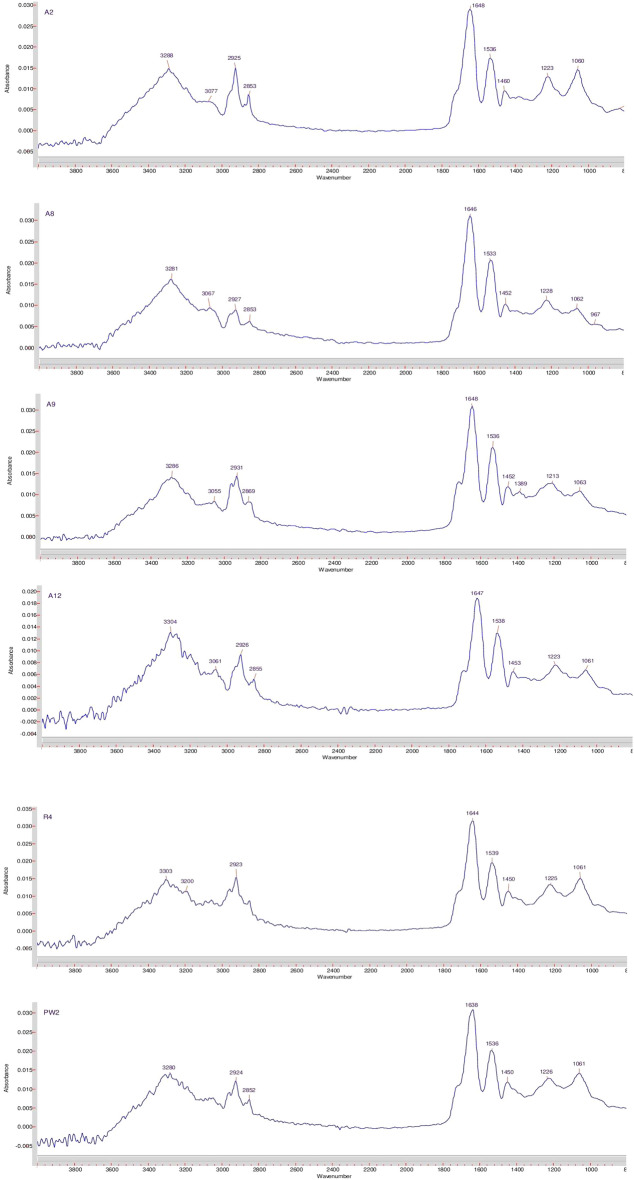
FT-IR absorption spectra of biosurfactants produced by *Bacillus* spp. strains isolated the Akingen field in West Kazakhstan.

We conducted analysis on all *Bacillus* spp. strains under study to elucidate the chemical structure of their biosurfactants. Infrared spectroscopy was employed to obtain spectra indicating the presence of peptide and aliphatic components in the biosurfactants of six strains (Fig. 7).

FT-IR spectra of biosurfactants produced by *Bacillus* spp. strains under study exhibited a characteristic band at 3288 cm^−1^, 3281 cm^−1^, 3286 cm^−1^, 3304 cm^−1^, 3303 cm^−1^, and 3280 cm^−1^, indicating the presence of the –NH bond (Fig. 7). Bands at 1648 cm^−1^ and 1536 cm^−1^ for the A2 strain, 1646 cm^−1^ and 1533 cm^−1^ for the A8 strain, 1648 cm^−1^ and 1536 cm^−1^ for the A9 strain, and 1647 cm^−1^ and 1538 cm^−1^ for the A12 strain, 1644 cm^−1^ and 1539 cm^−1^ for the R4 strain, 1638 cm^−1^ and 1536 cm^−1^ for the PW2 strain suggested the presence of the -CO–N bond. Meanwhile, bands at 2927–2853 cm^−1^ and 1452–1228 cm^−1^ reflected the stretching vibrations of (–CH) CH2 and CH3 groups, respectively, in aliphatic chains of the A8 strain. Similar results were observed for other *Bacillus* spp. strains. The absorption peak at 1730 cm^−1^ indicated the presence of complex ester carbonyl groups (–CO) bond.

The results indicate the presence of both peptide and aliphatic components in the biosurfactants of all *Bacillus* spp strains under study. These characteristics confirmed their lipopeptide nature, which could be significant for their potential applications in MEOR.

## Discussion

Several parameters, such as residual oil saturation, hydrocarbon composition, the chemical composition of fluids, reservoir depth, temperature, salinity, estimated net oil gain, and economic aspects, must be considered when extracting residual oil using MEOR processes^22^. Indeed, MEOR efficiency depends on many factors, but the reservoir’s salinity, temperature, and pH correspond to the most important ones. For instance, according to Sayyouh et al.^23^, in most reservoir selection criteria, the upper limit of salinity measured as a total dissolved solids (TDS) was 100,000 ppm. Meanwhile, She et al.^2^ reported that two microorganisms isolated from the produced fluid grew well in the salinity range from 100,000 to 200,000 ppm. However, when the salinity was higher than 200,000 ppm, these microorganisms grew much slower. On the other hand, Safdel et al.^21^ based on investigations of various sources, found that the salinity of the formation water ranged from < 10 g/l to more than 273 g/l. Guo et al.^24^ suggested reservoirs with temperatures from 20 to 80 °C are suitable for MEOR, yet the optimal temperature is 30–60 °C. Meanwhile, the pH of oil is in the range of 3–10. For the development of microorganisms, pH should be close to neutral^25^. The salinity, temperature, and pH value of the formation water tested in our study are close to the above criteria (Table 1). Therefore, the oil tanks we chose are applicable for MEOR since they showed optimal parameters.

For successful MEOR, it is necessary to apply microorganisms well adapted to the specific oil environments, e.g., isolated and cultivated in wells^26^. Indeed, (i) the oil spreading test, (ii) the emulsifying activity, and (iii) stalagmometric methods are often incorporated during searching for bacteria with potential usage in MEOR^27,28^. We also applied these approaches to test the isolated bacteria to identify potential biosurfactant producers (Table 2). As suggested by Simpson et al.^29^, in the oil spreading test, we included as a reference the RS-1 strain, which typically produces diameter clear zone of about 2.6 cm. Moreover, when testing the emulsifying activity, we followed the recommendation of Bosch et al.^30^, that an emulsification index of 50% or more defines the emulsion as stable. Nine our isolates, A2, A4, A8, A9, A11, A12, R1, R4, and PW2, met the requirements of the above criteria. It is generally accepted that MEOR-promising microorganisms should reduce the surface tension of the liquid medium by 40 mN/m or less^31^. Various studies have found that the surface tension is linked to the production of lipopeptides, e.g., by *Bacillus* strains can be reduced even to 28–24 mN/m^32–37^. In our study six isolates, e.g. A2, A8, A9, A12, R4, and PW2, showed the best results for the surface tension (< 40 mN/m), but the A9 isolate demonstrated the most promising surface tension (32.76 ± 0.3 mN/m).

All the oilfield isolates selected for potential usage in MEOR were classified into the genus *Bacillus*^38^, specifically to the *B. subtilis* group^39^. Interestingly, on one hand, the biochemical profiling showed that oil-associated *Bacillus* spp. belonged to the *B. subtilis* group, and on the other hand, indicated their diverse metabolic pathways and biochemical heterogeneity. Although *Bacillus* spp. are considered aerobic microorganisms, they also grow well in the limited presence of oxygen, which can be noted in oil wells^38^. Moreover, due to their ability to form endospores, representatives of *Bacillus* spp. are also resistant to stressful changes in environmental conditions that are inevitable when microorganisms enter the oil reservoir from the surface^40^. Therefore, *Bacillus* species are suitable candidates for MEOR by stimulating indigenous bacterial species or by industrially producing exogenous inoculation for injection into a reservoir^41,42^.

From perspectives of MEOR application, the *Bacillus* species are most studied producers of biosurfactants, biopolymers, and biomass for selective plugging^3^. Biosurfactants synthesized by *Bacillus* spp. are mostly lipopeptides, such as surfactin and lichenysin. These biosurfactans produced by *B. subtilis* and *B. licheniformis* are reported to have high surface activity beneficial for MEOR^35^, e.g., by reducing the surface and interfacial tension between oil and water^8,43^. Moreover, *B. subtilis* and *B. licheniformis* are known as producers of the levan biopolymer, which exhibits a stronger effect on permeability^44^. Effective permeability is also reduced due to the growth of bacteria biomass^45,46^. Indeed, biosurfactants produced by *Bacillus* spp. may recover up to 60% of the residual oil in sand-packed columns^47,48^.

*Bacillus* spp. isolated from the Akingen oilfield possessed the surfactin operon (*srfAA*, *srfAB*, *srfAC*, *srfAD*), that encodes the subunits of the surfactin synthetase^49^. Although Płaza et al.^50^ identified *srfAA* in both, *B. subtilis* and *B. licheniformis*, we found neither the gene in *B. licheniformis* nor in *B. paralicheniformis.* Meanwhile, Xu et al.^51^ in a whole-genome investigation of 47 *Bacillus* spp. strains, found three genes of the *srf* operon (*srfAA*, *srfAB*, and *srfAC*) in *B. safensis*, *B. subtilis*, *B. licheniformis*, and *B. paralicheniformis*. In addition, the authors noted incomplete the *srf* operon with missing genes in some strains. Similar results were also found in our study (Table 4). The observed phenomenon indicates that the *srf* operon undergoes complex and dynamic processes of evolution involving gene duplications, losses, rearrangements, and horizontal gene transfers^51,52^.

In contrast to Madslien et al.^53^, who proved that the *lch* genes are widely present in *B. licheniformis*, we only found the *lchAA* gene in one isolate, *B. licheniformis* PW2 (Table 4). Interestingly, the *sacB* gene encoding levan was presented in four out of six strains. Indeed, *B. subtilis* and *B. licheniformis* are well recognized for producing effectively levan^44,54,55^.

In order to have a broader view on the potential production of biosurfactants by the oilfield isolates, we assessed the relative expression of the biosurfactants genes among the *B. subtilis* group isolates. There is clear variability in the expression of the *srf* operon and the *sacB* gene between the strains. Moreover, the temperature of the culture moderately modifies the level of relative expression of these genes, with the maximum level recorded at 40 °C. On the other hand, increasing the salinity of the substrate in the range of 1–10% results in a radical increase in the expression of the studied genes.

Crude oil can be employed as a carbon source for biosurfactant production, and various studies have demonstrated the potential of different bacterial strains for this purpose. Based on the findings of Purwasena and colleagues^56^, the *B. licheniformis* DS1 strain exhibits biosurfactant production with a yield of 0.4 g/L, utilizing crude oil as a carbon source. Similarly, *B. subtilis* DM-04 exhibits a biosurfactant yield of 0.65 g/L, also when employing crude oil as the carbon source, in accordance with the findings of Das and Mukherjee^57^.

The stretching modes observed in the wavenumber range for -NH, -CO–N, and -CO bonds, as well as the -CH_3_ and -CH_2_ fractions, align with findings previously reported by De Faria et al^58^. Within the biosurfactant spectrum of *B. subtilis* strain LSFM-05, identifiable bands signaling the presence of a peptide component were detected. Specifically, wavenumber values of 1650 cm^−1^ (indicating the stretching of the CO–N bond) and 1540 cm^−1^ (reflecting the deformation mode of the N–H bond in conjunction with the stretching of the C–N bond) pointed to the existence of peptides. The presence of an aliphatic chain was discerned through C–H modes falling within the ranges of 2970–2850 cm^−1^ and 1450–1380 cm^−1^. Furthermore, an absorption band at 1730 cm^−1^ corresponded to the carbonyl group of an ether.

In the study by Zhang et al.^59^, Fourier-transform infrared spectra of biosurfactants derived from *B. atrophaeus* 5-2a revealed a distinct band at 3308.28 cm^−1^, indicating the presence of the –NH bond. Bands at 1652.40 cm^−1^ and 1540.97 cm^−1^ suggested the existence of the –CO–N bond, while bands at 2959.92–2928.66 cm^−1^ and 1456.85–1387.09 cm^−1^ represented the stretching (–CH) of CH2 and CH3 groups, respectively, within aliphatic chains. The absorption peak at 1736.07 cm^−1^ indicated the presence of ether carbonyl groups (the –CO bond).

Additionally, Joshi et al.^60^ demonstrated that the biosurfactant spectrum from *B. subtilis* 20B showcased characteristic bands confirming the presence of peptides. Specifically, wavenumber values of 3305 cm^−1^ (indicating the stretching of the NH bond) and 1643 cm^−1^ (indicating the stretching of the CO–N bond) signaled the existence of peptides. Bands within 2956–2924 cm^−1^, 2869 cm^−1^, 1463 cm^−1^, and 1377 cm^−1^ provided evidence of aliphatic chains (–CH3, –CH2–) in the composition. A prominent band at 1734 cm^−1^ indicated the presence of a carbonyl group.

## Materials and methods

### Sample collection

Formation water samples were randomly collected from active oil wells (No. 302, No. 329) and block cluster pumping station (output) in the Akingen field in West Kazakhstan (Fig. 1). Water samples were stored in 500 ml sterile plastic bottles, kept in a temperature controlled cool box (4–8 °C), and transferred within 24 h to the laboratory for further study.

### Chemical characterization of the samples

The chemical characteristics of samples were investigated according to the government standards (GOST standards). HCO_3_^−^ ions, CO_3_^−^ ions, and pH were determined by an electrometric method, whereas suspended substances and salinity were assessed by a gravimetric method according to GOST 26,449.1-85^61^. The SO_4_^2−^, Cl^−^, Ca^2+^, Na^+^  + K^+^, Mg^2+^, and NO_3_^−^ ions were analyzed by gravimetric, mercurimetric, complexometric, computational, and ion chromatography methods according to GOST 26,449.1-85^61^. Iron ions were determined by the complexometric method according to GOST 23,268.11-78^62^. Iodide and bromide ions were detected by ion chromatography, iodometric titrimetric, and titrimetric according to GOST 23,268.16-78^63^, GOST 23,268.15-78^64^. The viscosity was determined using a viscometer according to the Republic of Kazakhstan ASTM D 445-2011 standard^65^. The density was determined using a hydrometer according to GOST 18,995.1-73^66^.

### Isolation of bacteria associated with oil

Isolation of bacteria from formation water samples was carried out using nutrient agar (NA) (HiMedia Laboratories, Mumbai, India). 0.1 ml of the sample was plated on NA plates, spread on the medium surface with sterile plastic sticks, and incubated at 40 °C under aerobic and anaerobic conditions. After three days of incubation, individual bacterial colonies of various morphologies were inoculated onto NA plates and after a day of cultivation at 40 °C, transferred to liquid nutrient broth (NB) (HiMedia Laboratories) containing glycerol (15% w/v) and finally stored in a freezer at − 18 °C.

### Screening for biosurfactant-producing isolates

Stone mineral salt solution (SMSS) was used for cultivation and screening of the biosurfactant-producing bacteria (g/l): 2.5 g NH_4_NO_3_, 0.5 g MgSO_4_ × 7H_2_O, 0.2g MnCl_2_ × 4H_2_O, 0.5 g CaCO_3_, 1 g Na_2_HPO_4_ × 7H_2_O, 0.5 g KH_2_PO_4_, 1% yeast extract, 2% sterile crude oil filtred with 0.2 µm Filtropur V50 (Sarstedt Inc., Newton, USA)^56^. Evaluation the surface activity with oil spreading test, stalagmometric method, and emulsification activity are simple methods suitable for first and qualitative screening of biosurfactant-producing microorganisms^31^. The primary detection of the presence of biosurfactant-producing isolates was carried out with the use of (i) oil spreading, (ii) emulsification activity, and (iii) stalagmometric methods. The oil spreading test was performed following the strategy proposed by Youssef and co-workers^67^. Emulsifying activity (E24) was executed by the method of Cooper and Goldenberg^68^, while the stalagmometric properties were measured with Traube’s stalagmometer^27^. All bacterial isolates were cultivated in 100 ml of SMSS medium with 2% sterile crude oil as a carbon source and were grown at a temperature of 40 °C for 72 h. 1% suspension of surfactant Tween-20 was used as a control. Cell-free microbial supernatant was used in all methods. The cell-free supernatant was collected by centrifuging at 8,000×g for 20 min. Further, the surface tension was determined by the Du-Nouy-Ring method in a tensiometer (ST1 power tensiometer) of selected bacterial isolates^31^. In this method, distilled water was taken as a control, and a total value of 6 repetitions were applied.

### Phenotypic identification of the biosurfactant and biopolymer producing bacteria

The isolates selected during the biosurfactant and biopolymer screening tests were inoculated onto nutrient agar (NA) (HiMedia Laboratories), incubated overnight at a temperature of 40 °C, and examined under phase-contrast microscopy (Olympus BX61). The preliminary classification of the isolates into the genus *Bacillus* was completed with the determination of their biochemical profiles assessed with an API 50 CH test and partially in an API 20 E test (BioMerieux, Marcy-I’Etoile, France) as described by Logan and Berkeley^39^.

### Genomic DNA isolation

Bacterial isolates were grown at 40 °C for 24 h on LB agar (Oxoid, Basingstoke, Hampshire, UK). Then, one single colony of each strain was inoculated in 3 ml of LB broth (Oxoid) and incubated overnight at 40 °C under vigorous shaking. After the incubation, the bacteria were centrifuged at 3,000 × g, and the cells were separated with acid-washed glass beads 425–600 µm (Sigma-Aldrich Com., Germany). Genomic DNA was extracted with DNeasy Blood and Tissue Kit (Qiagen GmbH, Hilden, Germany) with the manufacturer’s protocol for Gram-positive bacteria. The DNA was suspended in double-distilled and nuclease-free water. The concentration and purity of the DNA were assessed using a NanoDrop 2000 spectrophotometer (ThermoFisher, Wilmington, USA). The DNAs were stored at − 20 °C for further use.

### Genetic identification

The genetic identification of bacteria was based on the determination of the direct nucleotide sequence of the 16S rRNA gene fragment, followed by the assessment of the nucleotide identity with sequences deposited in the GenBank. A 777 bp fragment of the 16S rDNA gene was amplified in PCR performed with UnF and UnR primers^69^ listed in Table S1 (Supplementary Material) in a total volume of 20 µl. The PCR mixture contained 150 ng of DNA, 1 unit of Maxima Hot Start Taq DNA Polymerase (ThermoFisher Scientific), 0.2 mM of each dNTP, 1X PCR buffer (ThermoFisher Scientific), 2.5 mM MgCl_2_, and 10 pmol of each primer. The PCR amplification program included prolonged denaturation at 95 °C for 7 min.; 30 cycles: 95 °C for 30 s., 55 °C for 40 s., 72 °C for 1 min.; final elongation for 7 min. at 72 °C. PCR was performed using the BioRad T100 amplifier (BioRad Laboratories Inc., Hercules, USA). The PCR results were analyzed by electrophoresis in a 1% agarose gel, followed by MIDORI Green Advance (Nippon Genetics Europe) staining and UV visualization. GeneRuler 1 kb DNA Ladder was used as a marker (ThermoFisher Scientific). Purification of PCR products from non-bound primers was carried out by the enzymatic method using exonuclease I (ThermoFisher Scientific) and alkaline phosphatase Fastap (ThermoFisher Scientific) according to Clarridge^70^. The sequencing PCR was performed using BigDye® Terminator v3.1 Cycle Sequencing Kit (Applied Biosystems, Waltham, USA) according to the manufacturer's instructions, followed by the separation of fragments on an automatic genetic analyzer 3730xlDNAAnalyzer (Applied Biosystems). The nucleotide sequences of the 16S rRNA gene were analyzed and combined into a common sequence in the SeqMan software (Applied Biosystems). After that, the end fragments (nucleotide sequences of primers, and fragments having a low-quality index) were removed. This allowed us to obtain a nucleotide sequence with a length of more than 650 bp, which was identified in GenBank using the BLAST algorithm (http://www.ncbi.nlm.nih.gov).

### Phylogenetic analysis

The alignment of nucleotide sequences was performed using the MUSCLE algorithm in the Mega platform ver. 11. The 16S rRNA gene sequences of the isolates and the reference strains were applied to construct the phylogenetic tree using the Neighbor-Joining (NJ) method^71^ together with MEGA11 software^72^. This method was chosen and applied based on the lowest score of Bayesian information criterion (BIC value). Branch quality was evaluated using 1,000 bootstrap replicates.

### PCR detection of the biosurfactant and biopolymer genes

Screening for the presence of surfactin, lichenysin, and levan genes was carried out using the primers listed in Table S1 (Supplementary material). Primer sequences were found in the NCBI database using Primer-BLAST. The PCR reaction was performed in a final volume of 12 µl containing 6.25 µl StartWarm HS-PCR Mix (A&A Biotechnology, Gdynia, Poland), 1.0 µl forward primer, 1.0 µl reverse primer, 2.75 nuclease free sterile H_2_O (A&A Biotechnology) and 1 µl DNA of each strain. Thermal cycling was carried out in the Agilent Technologies SureCycler 8800 (Agilent Technologies, USA) with an initial denaturation of 95 °C for 5 min followed by 30 cycles at 95 °C for 1 min, 56–64 °C at 1 min, and 72 °C for 1 min, followed by a final extension of 72 °C for 5 min. Each amplification product was analyzed by electrophoresis using a 1% agarose gel followed by MIDORI Green Advance (Nippon Genetics Europe) staining and UV visualization. GeneRuler 1 kb DNA Ladder was used as a marker (ThermoFisher Scientific).

### RNA isolation, reverse transcription, and qPCR analyses

The biosurfactant and biopolymer-producing *Bacillus* spp. isolated in this study from oilfield-associated samples were grown in brain heart infusion broth (Oxoid) at temperatures of 20 °C, 30 °C, 40 °C, and 50 °C, and at various concentrations of sodium chloride (10, 50 and 100 g/l) at 40 °C (Sigma-Aldrich). Total RNA was isolated from overnight cultures of the strains, using the Total RNA Mini Plus Kit (A&A Biotechnology) according to the manufacturer’s protocol as described previously^73^. The quantity and purity of RNA were assessed using the NanoDrop 2000 Spectrophotometer and electrophoresis in a 1% agarose gel followed by MIDORI Green Advanced staining and UV visualization. For the synthesis of cDNA, reverse transcription was performed using High-Capacity cDNA Reverse Transcription Kit (Applied Biosystems, Foster City, USA) in a Veriti 96 Well thermal cycler (Applied Biosystems) with the thermal protocol as follows: 10 min at 25 °C, 120 min at 37 °C, 5 min at 85 °C, and 5 min at 4 °C. Finally, all the cooled samples were used as templates for qPCR. Real-time amplifications were carried out in a StepOne Plus thermocycler (ThermoFischer Scientific). These reactions with a final volume of 20 µl were performed in thin-walled optical tubes containing 10 µl of RT 2 × PCR MasterMix A Kit (A&A Biotechnology), 1 µmol of each primer, 2 µl of cDNA, and 6 µl of sterile, nuclease-free water. Condition of amplification are given in Supplementary material Table S1. An analysis of melting curve followed each amplification. In this study, primers designed in the Primer3 Output database were used (Supplementary material Table S1). Potential changes in relative expression were calculated according to Pfaffl’s model^74^ with the 16S rRNA gene as an endogenous control. It was confirmed that the expression of this gene is similar in the whole range of tested media and temperatures.

### Crude biosurfactant isolation and chemical characterization

Biosurfactants were extracted using the acid precipitation method outlined by Zhang et al., Nitschke and Pastore ^59,75^. Cell free supernatant was harvested through centrifugation at 8,000×g for 20 min. In a nutshell, the cell-free supernatant was adjusted to pH 2.0 with 6M HCl and left overnight at 4 °C to ensure complete biosurfactant precipitation. The resulting precipitate was gathered by centrifugation at 8,000×g for 20 min and underwent two rinses with water adjusted to an acidic pH of 2.0. The unrefined biosurfactants were dried in a drying cabinet at 110 °C for 24 h and weighed.

The FT-IR spectrum of the dried biosurfactants were obtained on a FT-IR spectrophotometer (Carry 660 Agilent, USA) in a dry atmosphere. The FT-IR spectra were collected in the range of 400–4000 wavenumbers (cm^−1^).

### Repositories

The GenBank accession numbers for the 16S rRNA gene sequence of the bacteria isolated in this study are as follow: *Bacillus safensis* subsp*. safensis* strain A2, OP565012; *Bacillus subtilis* strain A8, OP565013; *Bacillus subtilis* strain A9, OP565014; *Bacillus subtilis* subsp*. subtilis* strain A12, OP565015; *Bacillus paralicheniformis* strain R4, OP565016; *Bacillus licheniformis* strain PW2, OP565017.

## Conclusion

The wells of the Akingen oil field in Western Kazakhstan are good candidates for applying of the MEOR technologies. Screening for surface tension abilities allowed the selection of six isolates from the reservoir waters of the field, which were classified as *Bacillus safensis subsp. safensis* strain A2*, Bacillus subtilis* strain A8, *Bacillus subtilis* strain A9, *Bacillus subtilis* subsp. *subtilis* strain A12, *Bacillus paralicheniformis* strain R4, and *Bacillus licheniformis* strain PW2, were identified as potential producers of biosurfactants. From this perspective, they are promising bacteria (especially A9) for use in oil recovery and more effective extraction of oil from reservoirs. However, it is necessary to study them in detail before further MEOR applications.

### Supplementary Information

Supplementary Table 1.

Supplementary Table 2.

Supplementary Figure 1.

## Data Availability

All data generated or analyzed during this study are included in this published article and its supplementary information files.
