# Supplementary material for: Biosurfactant and biopolymer producing microorganisms from West Kazakhstan oilfield
Source: Sci Rep. 2024 Jan 27;14:2294. doi: 10.1038/s41598-024-52906-7 (PMC10821952; doi:10.1038/s41598-024-52906-7)
Supplement: Supplementary file 1 — Supplementary Table 1. [file 41598_2024_52906_MOESM1_ESM.pdf]

Biosurfactant and biopolymer producing microorganisms from West Kazakhstan oilfield

Ulzhan Shaimerdenova, Gulzhan Kaiyrmanova, Wioleta Lewandowska, Marek Bartoszewicz, Izabela Swiecicka, and Aliya Yernazarova

Table S1. Primers used in the study.

Product	Gene	Sequence	PCR product size (bp)	Annealing temperature (°C)
Primers used for detection of the 16S rRNA gene				
16S rRNA	*16S rDNA*	UnF: 5'-GAGTTTGATCCTGGCTCAG-3'<br>UnR: 5'-GGACTACCAGGGTATCTAAT-3'	777	55
Primers used for detection of the biosurfactants and biopolymer genes				
Surfactin	*srfAA*	F: 5'-AAGGGCCATTGCCAATACGA-3'<br>R: 5'-ACTTTGCCGTTTGCCGTAAC-3'	501	58
	srfAB	F: 5'-GTCACGAATCATTGCGGACG-3'<br>R: 5'-GTAACGGAGCCGATGACGAT-3'	631	58
	srfAC	F: 5'-CGGAAAGCCGATCAGCAATG-3'<br>R: 5'-CTCCGAGCGCAAAGAAATCG-3'	665	58
	srfAD	F: 5'-GGATCTCGAAGAGCTGACGG-3'<br>R: 5'-CTTCTTCCACCCTTCCGCAT-3'	430	58
Lichenysin	*lchAA*	F: 5'-TGAACGGCACAAAATGCAGG-3'<br>R: 5'-CGTTTGATCGATTCGCGCTT-3'	678	60
Levan	*sacB*	F: 5'-TGTCGCAAACTATCACGGCT-3'<br>R: 5'-TCAATCATACCGAGAGCGCC-3'	663	64
Primers used for qPCR				
Surfactin	*srfAB*	F: 5'-GTCAAAACAGCCAGCCTTCA-3'<br>R: 5'-TATCCTCTTCACGCTCGACC-3'	241	60
	srfAC	F: 5'-CCGGGAGATGAGATGCTGAT-3'<br>R: 5'-GCGTTCACTGCTTCCTTGAA-3'	248	60
Levan	*sacB*	F: 5'-ACCTTTACTACCGCACTGCT-3'<br>R: 5'-TTTGTAATGGCCAGCTGTCC-3'	235	57

[a] F, forward primer; R, rivers primer.
