# Supplementary material for: Biosurfactant and biopolymer producing microorganisms from West Kazakhstan oilfield
Source: Sci Rep. 2024 Jan 27;14:2294. doi: 10.1038/s41598-024-52906-7 (PMC10821952; doi:10.1038/s41598-024-52906-7)
Supplement: Supplementary file 2 — Supplementary Table 2. [file 41598_2024_52906_MOESM2_ESM.pdf]

Table S2. Biochemical profiles of the *Bacillus* spp. isolates from formation water samples from active oil wells (No. 302, No. 329) and block cluster pumping station (output) in the Akingen field in West Kazakhstan.

System	Test	Active ingredients	Strain					
			A2	*A8*	*A9*	*A12*	*R4*	*PW2*
API 20 E	ONPG	2-nitrophenyl-βD-galactopyranoside	+	+	+	+	+	+
	ADH	L-arginine	-	-	-	-	-	-
	LDC	L-lysine	-	-	-	-	-	-
	ODC	L-ornithine	-	-	-	-	-	-
	CIT	trisodium citrate	-	-	-	-	-	-
	H2S	sodium thiosulfate	-	-	-	-	-	-
	URE	urea	-	-	-	-	-	-
	TDA	L-tryptophan (tryptophan deaminase)	-	-	-	-	-	-
	IND	L-tryptophan (indole production)	-	-	-	-	-	-
	VP	sodium pyruvate	-	-	-	+	+	+
	GEL	gelatin	+	+	+	+	+	+
API 50 CH	control		-	-	-	-	-	-
	GLY	glycerol	-	+	+	+	+	+
	ERY	erythritol	-	-	-	-	-	-
	DARA	D-arabinose	-	-	-	-	-	-
	LARA	L-arabinose	+	+	+	+	+	+
	RIB	D-ribose	+	+	+	+	+	+
	DXYL	D-xylose	-	+	+	+	+	+
	LXYL	L-xylose	-	-	-	-	-	-
	ADO	D-adonitol	-	-	-	-	-	-
	MDX	methyl-β-D-xylopyranoside	-	-	-	-	-	-
	GAL	D-galactose	-	-	-	-	+	+
	GLU	D-glucose	+	+	+	+	+	+
	FRU	D-fructose	+	+	+	+	+	+
	MNE	D-mannose	+	+	+	+	+	+
	SBE	L-sorbose	-	-	-	-	-	-
	RHA	L-rhamnose	-	V	-	-	V	-
	DUL	dulcitol	-	-	-	-	-	-
	INO	inositol	-	+	+	+	+	+
	MAN	D-mannitol	+	+	+	+	+	+
	SOR	D-sorbitol	-	-	-	-	-	-
	MDM	methyl-α-D-mannopyranoside	-	-	-	-	-	-
	MDG	methyl-α-D-glucopyranoside	+	+	+	+	+	+
	NAG	N-acetylglucosamine	-	-	-	-	-	-
	AMY	amygladin	-	+	+	+	+	+
	ARB	arbutin	+	+	+	+	+	+
	ESC	esculin ferric citrate	+	+	+	+	+	+
	SAL	salicin	+	+	+	+	+	+
	CEL	D-cellobiose	+	+	+	+	+	+
	MAL	D-maltose	-	+	+	+	+	+
	LAC	D-lactose	-	-	-	+	-	-
	MEL	D-melibiose	-	+	+	+	+	+

	SAC	D-saccharose	+	+	+	+	+	+
	TRE	D-trehalose	+	+	+	+	+	+
	INU	inulin	-	+	+	-	+	+
	MLZ	D-melezitose	-	-	-	-	-	-
	RAF	D-raffinose	-	+	+	+	+	+
	AMD	starch (amidon)	-	+	+	+	+	+
	GLYG	glycogen	-	+	+	+	+	+
	XLT	xylitol	-	-	-	-	-	-
	GEN	gentiobiose	-	-	-	-	-	-
	TUR	D-turanose	-	+	+	-	+	+
	LYX	D-lyxose	-	-	-	-	-	-
	TAG	D-tagatose	-	+	+	+	+	+
	DFUC	D-fucose	-	-	-	-	-	-
	LFUC	L-fucose	-	-	-	-	-	-
	DARL	D-arabinose	-	-	-	-	-	-
	LARL	L-arabinose	-	-	-	-	-	-
	GNT	potassium gluconate	-	-	-	-	-	-
	2KG	potassium 2-ketogluconate	-	-	-	-	-	-
	5KG	potassium 5-ketogluconate	-	-	-	-	-	-
Note: «+» - Positive; «-» - Negative; «V» - Doubtful.								
