# Supplementary material for: Biosurfactant and biopolymer producing microorganisms from West Kazakhstan oilfield
Source: Sci Rep. 2024 Jan 27;14:2294. doi: 10.1038/s41598-024-52906-7 (PMC10821952; doi:10.1038/s41598-024-52906-7)

Biosurfactant and biopolymer producing microorganisms from West Kazakhstan oilfield

Ulzhan Shaimerdenova, Gulzhan Kaiyrmanova, Wioleta Lewandowska, Marek Bartoszewicz, Izabela Swiecicka, and Aliya Yernazarova

Figure S1. Agarose gel electrophoresis of PCR products for the genes of biosurfactants and biopolymer. M: GeneRuler 1 kb DNA Ladder. A) genes *srfAA* and *sacB*; B) genes *srfAC* and *srfAD*; C) gene *srfAB*; D) gene *lchAA*. 1 - *Bacillus safensis subsp. safensis* strain A2*; 2- Bacillus subtilis* strain A8; 3 - *Bacillus subtilis* strain A9, 4 - *Bacillus subtilis* subsp. *subtilis* strain A12, 5 - *Bacillus paralicheniformis* strain R4; and 5 - *Bacillus licheniformis* strain PW2.

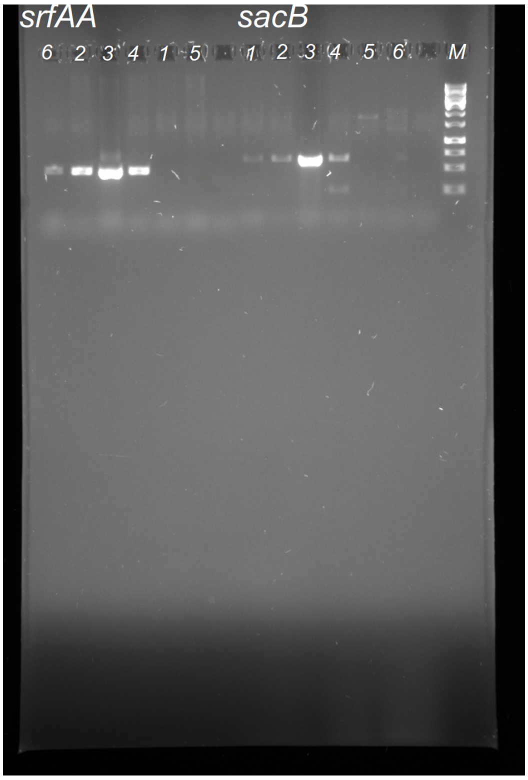

(A)

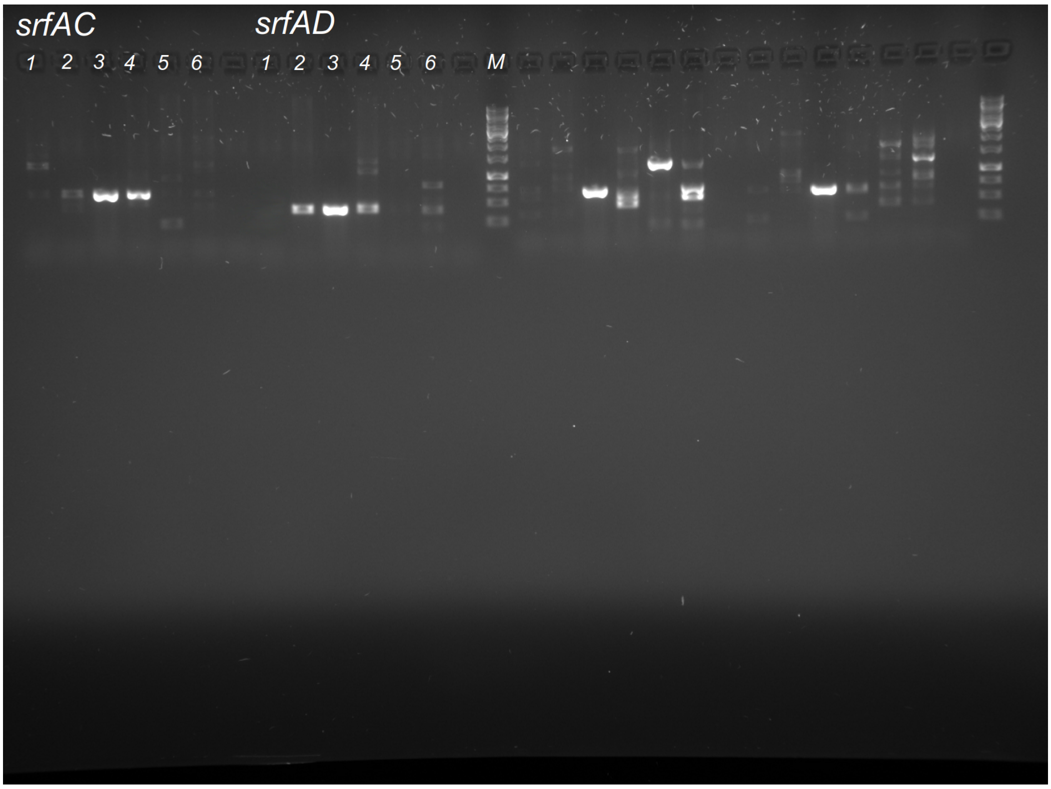

B)

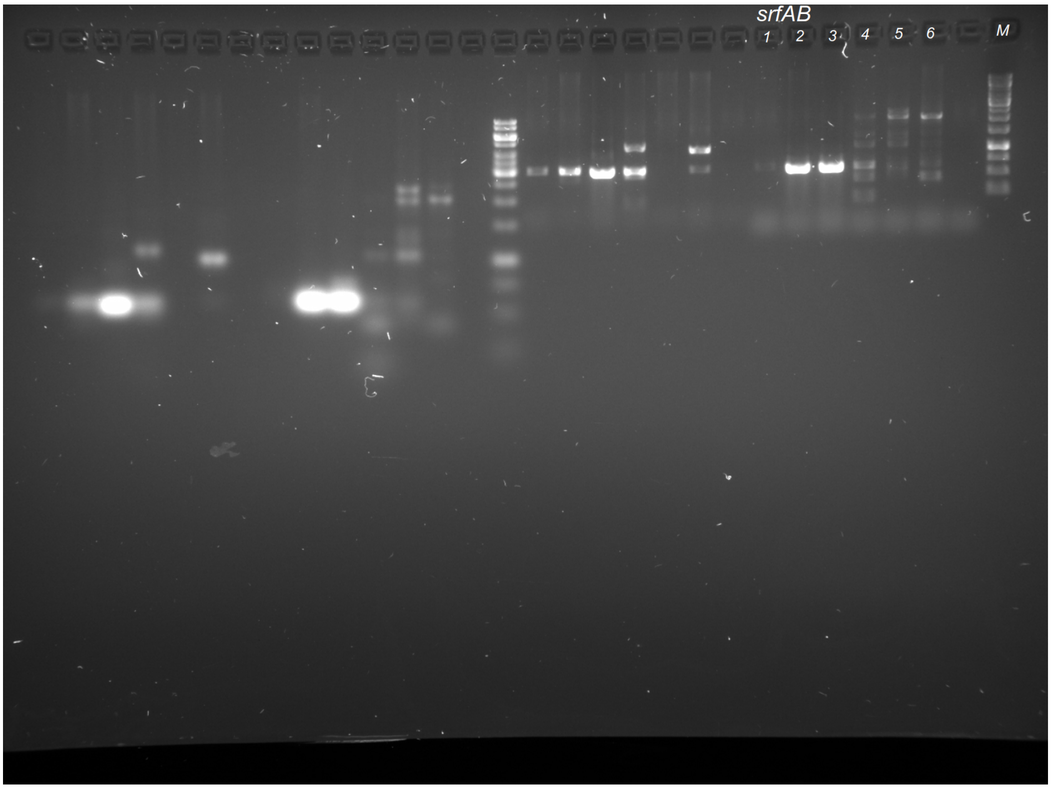

C)

D) 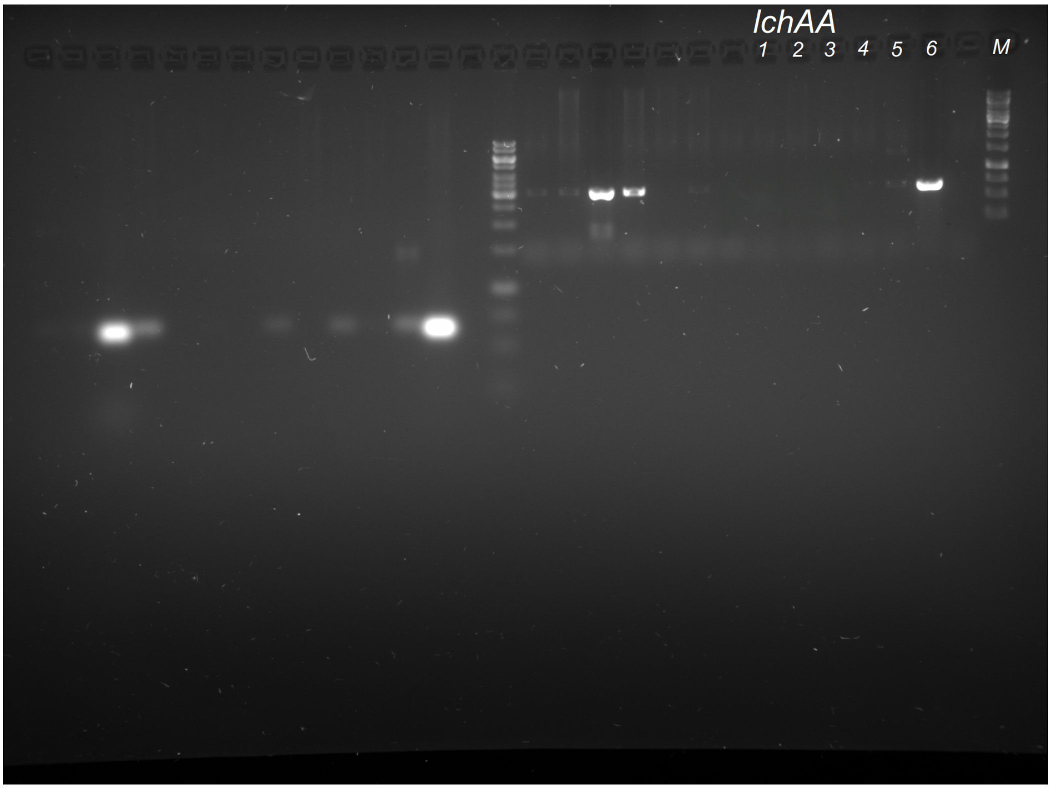

Supplement: Supplementary file 3 — Supplementary Figure 1. [file 41598_2024_52906_MOESM3_ESM.pdf]
